# Impact of stock health on fish prices: Evaluation and implications for food accessibility

**DOI:** 10.1371/journal.pone.0261580

**Published:** 2021-12-22

**Authors:** Marceliano Rodriguez, Domingo Calvo-Dopico, Estefanía Mourelle

**Affiliations:** 1 Department of Business, Facultade de Economía e Empresa, Universidade da Coruña, A Coruña, Spain; 2 Department of Economics, Facultade de Economía e Empresa, Universidade da Coruña, A Coruña, Spain; Universitat Jaume I, SPAIN

## Abstract

The continuous rise of the world’s population has made food security a major point of the global agenda, with fisheries providing a key source of nutrition, especially in developing countries. Ensuring their health is key to maintain the availability of the resource, but its effect over accessibility is yet unclear. In this paper, we discuss the relevance of stock health for ensuring the price accessibility of the resource. A Least Square Dummy Variable panel model is proposed for bluefin tuna prices, with a biological explanatory component, and dummy variables reflecting changes in fishing trends. Both have proven to be significant to explain annual price variations, with improvements in stock health achieving price reductions.

## 1 Introduction

According to FAO [1], on average 17% of our protein intake is fish-based, reaching higher numbers in developing countries. Nevertheless, seafood exploitation levels have gathered an important level of attention in recent decades. The estimation of underfished stocks has globally experienced a major fall, caused by an increase of fully exploited stocks and a significant increase in overfished ones [2]. There is a clear challenge for food security, particularly in the more vulnerable developing world [3, 4].

This challenge is twofold. One side is food availability, this is, ensuring the continuity of the resource in the long term, with responsible levels of exploitation that ensure healthy stocks. The other is accessibility, ensuring that consumers can afford it. The economic benefits of rebuilding stocks has been proven, as the income from increased efficiency, costs reductions and the elimination of public subsidies outweighs the costs of the process in the long term [5, 6]. Just as the sustainable management of fishery resources is key to providing benefits to stakeholders, ensuring safe and accessible food sources is of utmost importance for nutrition [7] and economic equality [8]. The question that we tackle with this paper lies within this area, particularly, how policies aimed at sustainable and environmentally respectful exploitation can increase the price accessibility of fishery resources. Our main contribution is the introduction of a biological variable in an econometric model of prices, which measures the previously stated effect.

Price models often include variables like market quantity [8–10], type of gear [9, 11], processing level [11], whether the product is fresh or frozen [12], quality [11, 13], fish characteristics, seasonality [14], buyers preferences [14, 15], or whether the species is a luxury one, in which case it will be influenced by seasonality and economic cycles [8]. Our proposal in this paper is to build a price model that includes a variable that reflects the sustainability of the exploitation of a fishery. In the same way that biological models have benefited from the use of economic components [16], the inclusion of biological variables in economic modelling offers interesting opportunities. Their importance in the field of fisheries economics has been ascertained by authors like Pincinato & Gasalla [17], who successfully employed similar variables like climate change to explain fish price volatility [18]. However, the use of biological variables in fisheries economic models has been quite scarce up till now. As far as we know, this is the first research that incorporates the health of the stock to explain variations in the price of fish. The inclusion of such variables will provide a more realistic view of price fluctuations not only with market trends, but also with the real situation of the stock, which could be extrapolated to similar studies in the field.

We will tackle the issue from an economic perspective with a panel data model, a Least Square Dummy Variable, LSDV, which will explain bluefin tuna market prices through market quantities, and a proposed biological variable reflecting the health of the stock. As trend changes in quantities and prices may hint ecological problems [17], the series have been studied for structural breaks, and when found, discussed and incorporated in the model through dummy variables. Generally, they reflect economic events (i.e. 2008 crisis) and milestones in the management of the fishery (i.e. TAC changes).

The paper is organized as follows. Section 2 provides a context of fisheries sustainability and management. Section 3 presents the databases and methodology employed. Section 4 is divided in three parts. The first part gives an overview of the fishery, while the second discusses trends and structural breaks on the series; the third part discusses the results of the model. Section 5 concludes.

## 2 Concepts and previous research

### 2.1 Overexploitation of fisheries and sustainability

**Fig 1:**
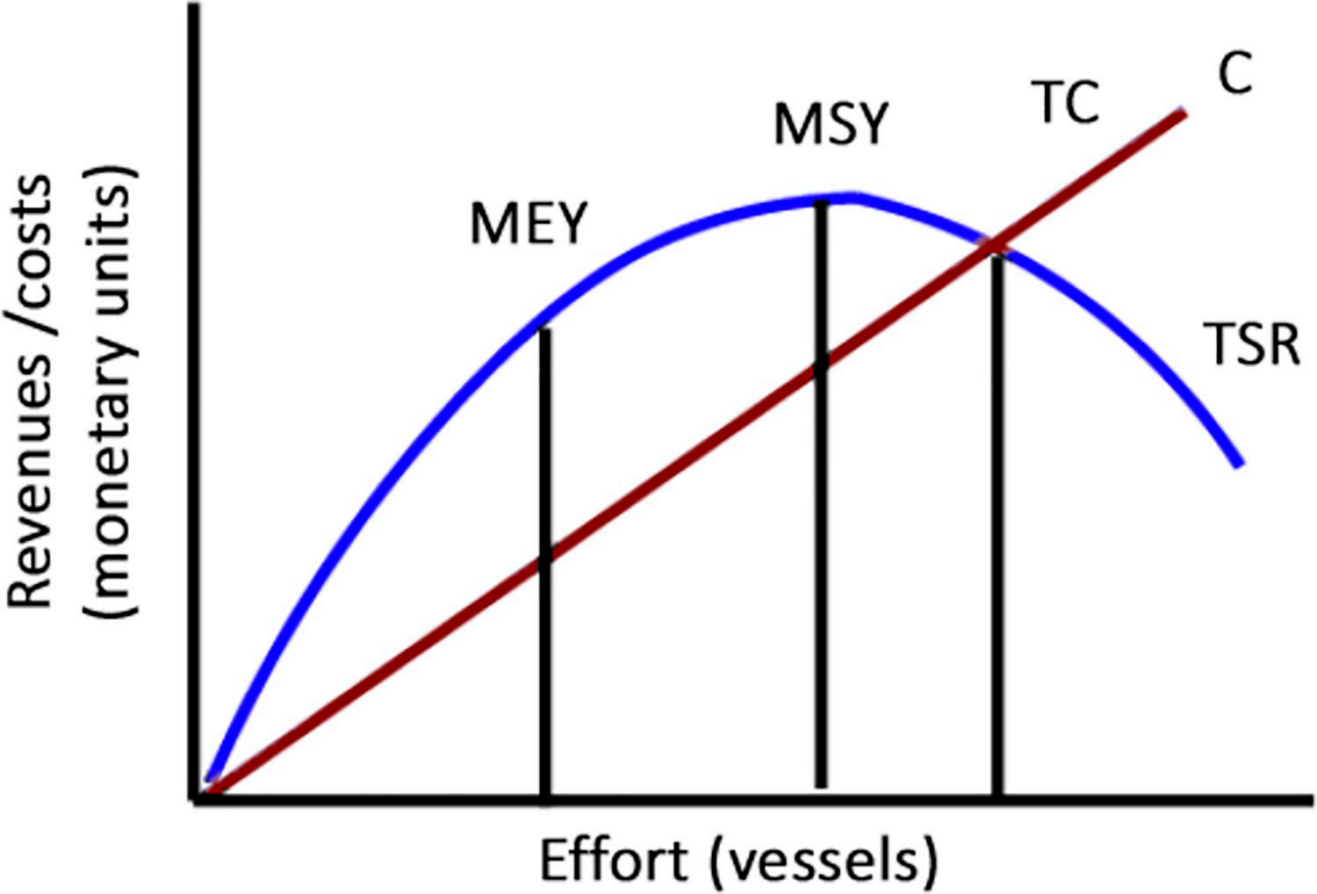
Gordon-Schaefer model. Own elaboration based on Seijo et al. [20].

Bioeconomic models relate the yield obtained from a fishery with its health and level of exploitation. For explaining the basic dynamics behind renewable resources, the Gordon-Schaefer model [19] is commonly used for its didactic and graphic nature. It is usually depicted with the following graph:

Assuming constant prices and costs, as seen in Fig 1 an underexploited fishery would provide increasing returns of scale when increasing fishing effort (usually seen as increasing the number of vessels). When fish population or the Spawning Stock Biomass (SSB) starts to decline, the increases in effort will start providing decreasing returns of yield/revenues, as the fishing grounds start to be saturated, but the stock is still capable of replenishing itself. Bellow the Maximum Sustainable Yield (MSY, the maximum point of exploitation without compromising the sustainability of the resource), the stock is no more able of replenishing, and its numbers start to decline.

**Fig 2:**
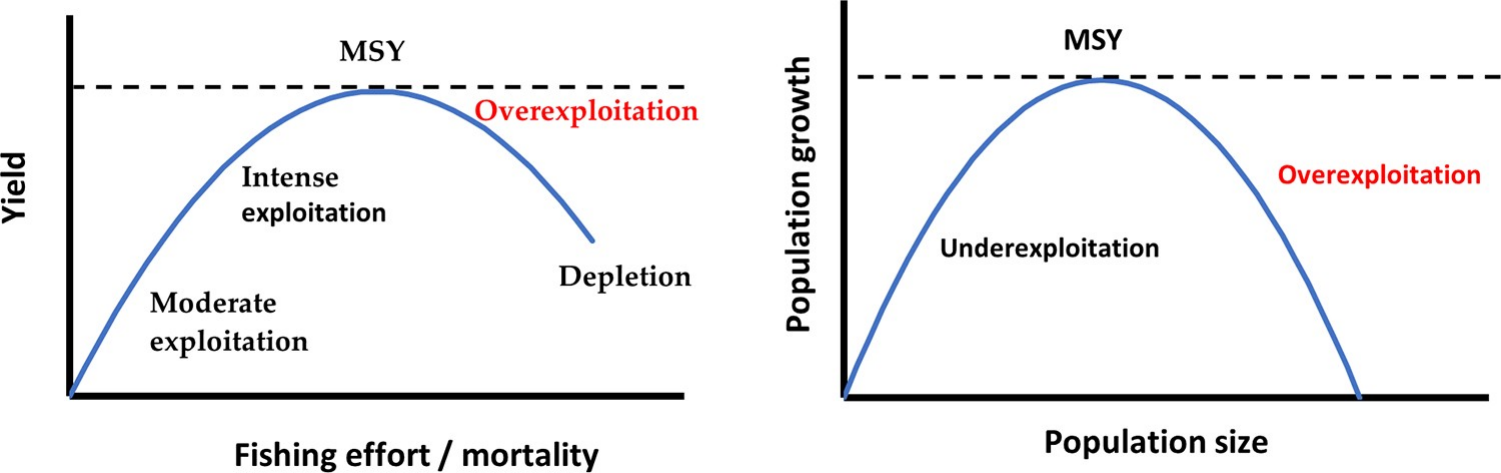
The MSY point. Own elaboration based on Seijo et al. [20].

A stock exploited at least at MSY level would maintain its production capability over time, while overstepping the MSY threshold would imply that the stock will reduce its yield [20]. As shown in Fig 2, MSY maximises stock growth, as the reproductive capabilities of the species are fully used, and the limiting effect of the environment is minimised. Therefore, as in Fig 2, MSY is the optimum level of exploitation to maximise long term yield. While MSY maximises the total yield, the limit between increasing and decreasing returns of scale would maximize the efficiency of the resources employed, and thus is known as Maximum Economic Yield (MEY), where final profit is maximised. If the fishing activity continues bellow the MSY, at some point the returns will not cover the costs, and the activity will not provide value.

This reference points are useful as management objectives and allow for the construction of indicators of the health of the stock–how sustainable the level of exploitation is-. A common and intuitive indicator is the coefficient between the current Spawning Stock Biomass and the correspondent value at a reference point–like MSY or MEY. In other words, this would be a coefficient between how many fish at reproductive age/size there are and how many there should be. We have selected this coefficient with MSY as reference point as an indicator of stock health because of its easiness of understanding and interpretation, and is easily available for multiple species and fisheries, through repositories like FishSource and RAM Legacy. The interpretation and modelling capability is simple, with values >1 entailing that the stock is exploited in a sustainable way, and <1 that the stock has no capacity to completely regenerate with the current level of exploitation.

With income and profit maximization points, there is a resemblance of the neoclassical economical idea of a self-regulated market equilibrium, in which stock health regulates the activity. However, scarcity generates price rises [9], acting as an incentive for overfishing and cancelling the equilibrium, resulting in Harding’s [21] idea that “unregulated resources tend to ruin”, and the need to manage the resource.

### 2.2 Managing stocks

From the above mentioned idea of the need of government intervention in the regulation of common resources [21], multiple authors like Acheson [22], Janssen *et al*. [23] and Garza-Gil *et al*. [24] have continued Ostrom [25] idea of integrative institutions that align the needs and objectives of management bodies with scientific advisors and fishermen. Involving fishers in management has been found key to ensure compliance with measures and to benefit from their knowledge of the resource. In line with this new idea, current Regional Fisheries Management Organizations (RMFOs) try not only to involve scientific bodies to study the stock and governments to enforce measures, but also fishers, to ensure their collaboration in the defence of the resource that defines their way of life. These institutions design and implement control measures, generally through the establishment of a global Total Allowable Catch (TAC), distributed among all countries involved, which are then responsible for distribution among their fleet. In general, we find successful cases of implementation of fisheries management strategies [26] and initiatives to engage and assist stakeholders [27]. However, there are also failed cases, such as the case of New England described by Rothschild *et al* [28] where management objectives were unclear and inadequate choice of model jeopardized resource improvement. Therefore, it is extremely important to carefully plan how the stock will be managed. This will be illustrated later with the case of Atlantic bluefin tuna, for which stakeholder participation proved to be key to the success of the management strategy.

Supranational institutions also play a key role in the movement towards sustainability. On the 80’s and 90’s decades, international institutions like the UN and specially UNCLOS started stressing and requiring sustainable fishery management, setting general standards and promoting the creation of RMFOS. From the 2.000 on, the focus has been put on establishing more specific standards and actions, and collaborating with the developing world and stakeholders [29]. In the EU, the regulations associated with the sustainability of marine and fisheries resources establishes that the sustainability of the species must be ensured in such a way that the reproduction of the fish stock is not endangered while maximizing captures [30]. In other words, fishermen must respect the maximum sustainable yield (MSY), no more fish should be caught than a given stock can produce in a given year.

While much progress has been made in recent years by managing institutions, they are limited by the fact that by involving multiple stakeholders, conflicts of interest arise among them, slowing down their action and jeopardising their objective of sustainability. As pointed by Stafford [31], while the interest of scientist is the sustainability of the fishing grounds, fishers seek economical sustainability, and local governments seek the benefit for their region. This effect is greater in the case of international fisheries, where stakeholders are spread through different countries, and various governments are involved [32]. Evidence of this is that Total Allowable Catches (TACs) are often set above scientific recommendations perhaps in interest of pressure groups instead of in the best interest for the whole. This will be observed later in the TAC and captures series of bluefin tuna.

## 3 Methodology

### 3.1 Data

Atlantic bluefin tuna, Thunnus thynnus, is currently one of the most prized commercial fishing species. Its population is divided into two sub-stocks, Western Atlantic and Eastern Atlantic—Mediterranean stock, with the last one being the most important. Currently, 27 countries harvest Atlantic bluefin tuna. The data employed in the analysis has been collected from the European Commission [33]; FAO [34]; ICCAT [35], and FishSource [36], and treated as follows.

To study the impact of stock health on the market, quantity and value data of international trade of Atlantic and Pacific bluefin tuna were collected from FAO [34] Fisheries commodities production and trade datasets. Prices were calculated by dividing the total value of all tuna products (aggregate of fresh, live and fresh/chilled) by total quantity and transformed to constant 2010 prices with World Bank [37] CPI data. As it appears that countries tend not to use the FAO product categories consistently among themselves and between different years, the study has employed the aggregated data to capture the variability of the whole value chain, and not be biased by reporting discrepancies.

For Atlantic stock status, FishSource [36] assessment stock health data has been employed. The coefficient between the SSB and the SSB at MSY has been chosen to reflect the biological status of the stock. As an indicator, it reflects in a clear and intuitive manner the current health of the stock. Values below one mean overexploitation, and values above 1 relate with sustainable managing and even underexploitation.

As FAO international trade database aggregates the statistics of Atlantic and Pacific bluefin tuna, the first decision has been to focus the study on the effect of the eastern Atlantic and Mediterranean stock of Atlantic bluefin tuna. Pacific stock was discarded due to scarcity and unreliability of stock status data. The criterion to make the country and time period selection was the maximisation of countries and time periods with available and homogeneous data. Also, to estimate the health effect of the Atlantic stock, just the countries with relevant captures have been included. Similarly, countries with a high trade in bluefin tuna but no relevant captures of Thunnus thynnus, such as China, have been excluded. From the initial sample of 27 countries currently exploiting the stock, 6 have been selected for the analysis with the above-mentioned criteria, representing 60% of captures. While higher numbers are desirable, the final sample provides a representative picture of the exploitation of Atlantic bluefin tuna. The chosen countries have been France, Italy, Japan, South Korea, Spain, and the United States.

Aiming for a balanced panel and homogeneous time series in terms of length, and due to data availability and missing values, the starting year for the analysis has been 1994. In addition, availability of data for the biological indicator, the coefficient between SSB and SSB_MSY_, have forced the quantities and prices series to end in 2011. While ICCAT [38] SCRS report indicates that the SSB is between sustainability boundaries and concludes that the fishery is not overfished (although with concerns over the possible effects of changing the recruitment assumptions in the VPA model), the last available observation for the ratio is in 2011, starting the current recovery trend.

### 3.2 The model

Panel models involve a combination of time series and cross-section data. As Wooldridge [39] has pointed out, unbalanced panels pose specification problems; for this reason, we put forward the use of balanced ones, with equal time periods T for each N cross section. Two of the most common specification techniques are fixed effects (FE) and random effects (RE). Fixed effects subtract the mean of each time period to obtain the time demeaned data; under strict exogeneity, the obtained estimator is unbiased [39]. The random effects estimator (RE) adds to FE the assumption of independence of all explanatory variables.

In this work we resort to a variation of the fixed-effects model, the Least-Squares Dummy Variable (LSDV) Regression Model, as this procedure considers the unobserved heterogeneity. In this case, the traditional OLS model incorporates dummy variables for each case or time effect [40]. The specification may also include differential slope dummies, i.e., the product of each dummy by the regressors. Thus, the expression of the model is as follows:

$$y_{it}=\alpha +\alpha_{1}D_{1i}+\cdots +\alpha_{n}D_{ni}+\beta_{1}X_{1it}+\cdots +\beta_{m}X_{mit}+\delta_{i}D_{1i}X_{1it}+\cdots +\delta_{k}D_{ni}X_{mit}+\mu_{it}$$ (1)

where n denotes the number of dummy variables, m the number of explanatory variables, k the number of interactions, t ranges from one to T, and i ranges from one to N.

Dummy variables can be individual-specific or period-specific, leading to a different value of the intercept for each cross-section or time period variable. By using this method, the residuals are homoskedastic and do no not show serial correlation, leading to consistent estimators. To estimate the model, the existence of unit roots in the series must be tested. For that purpose, the test derived by Levin, Lin & Chu [41] will be performed.

The proposed model will explain bluefin tuna trade prices with trade quantities, the health of the stock, and dummy variables to account for the structural breaks caused in some series by management events in the fishery. The first and main explanatory variable is trade quantity, as prices are initially set by auction traders based on it [10]. It is an exogenous variable that depends upon quotas, regulations, or stakeholders’ interests, among others [42], and shows a negative relationship with prices, as higher quantities (abundance) lower the prices, and vice versa [8, 9]. Quantities could be influenced by prices too, if the higher prices generated by scarcity act as an incentive to overfish, so we have tested the exogeneity of the variable though the Durbin–Wu–Hausman test [43]. As shown in Table 1, we do not reject the null hypothesis of exogeneity in the series. This follow [10] idea that fish prices depend upon quantities, as they are set by auction’s traders.

**Table 1:** Durbin–Wu–Hausman test for quantities.

Country	Asymptotic test statistic: Chi-square	p-value
Japan	0.0059	0.9388
Italy	2.2224	0.1360
France	3.5091	0.0610
Korea	0.0626	0.8024
Spain	0.4669	0.4944
USA	0.1183	0.7309

The second variable, the health of the stock, entails its ability to continue producing in the long term and will be reflected as the coefficient between the current reproductive biomass of the stock and the required to have a sustainable exploitation (that will continue in the long rank). Fist lags for quantities and health will be included, as fish price formation might be influenced by expectations from the past performance of the fishery. The last factor, the dummy variables, account for specific events in the history of the fishery, which would distort the series if not accounted for. They will be further explained in section 4.2.2.

## 4 Results and discussion

### 4.1 Overall picture

As shown in Table 2, the most important capturing countries are the EU (mainly Spain, France and Italy), Japan, and North African countries such as Morocco, Tunisia and Libya.

**Table 2:** Thunnus thynnus catches and quota per country.

	Captures 2016		Quota 2019	
Country	Tonnes	*%*	Tonnes	*%*
Spain	4.433	*21*.*7%*	5.532	*17*.*3%*
France	3.397	*16*.*6%*	5.459	*17*.*1%*
Italy	2.488	*12*.*2%*	4.308	*13*.*5%*
Japan	1.923	*9*.*4%*	2.544	*8*.*0%*
Morocco	1.783	*8*.*7%*	2.948	*9*.*2%*
Tunisia	1.461	*7*.*1%*	2.400	*7*.*5%*
Libya	1.368	*6*.*7%*	2.060	*6*.*4%*
Turkey	1.324	*6*.*5%*	1.880	*5*.*9%*
Others	2.257	*11*.*0%*	4.860	*15*.*2%*
	**20.434**	***100*.*0%***	**31.991**	***100*.*0%***

Own elaboration from European Commission [33], FAO [34] & ICCAT [35].

The two stocks are assessed and managed by the RMFO ICCAT (International Commission for the Conservation of Atlantic Tunas). This organisation has two purposes. On the one hand, to advise on the biological status of stocks with scientific data. On the other hand, to bring together all countries with economic interest in the fisheries, to make all the decisions relevant to the exploitation, one of the most vital, the TACs. Historically, high fishing pressure has been exerted over the stock. The increased catches between the 80s and the 90s raised concerns on its biological situation and sustainability, leading to the implementation of management plans by ICCAT.

**Fig 3:**
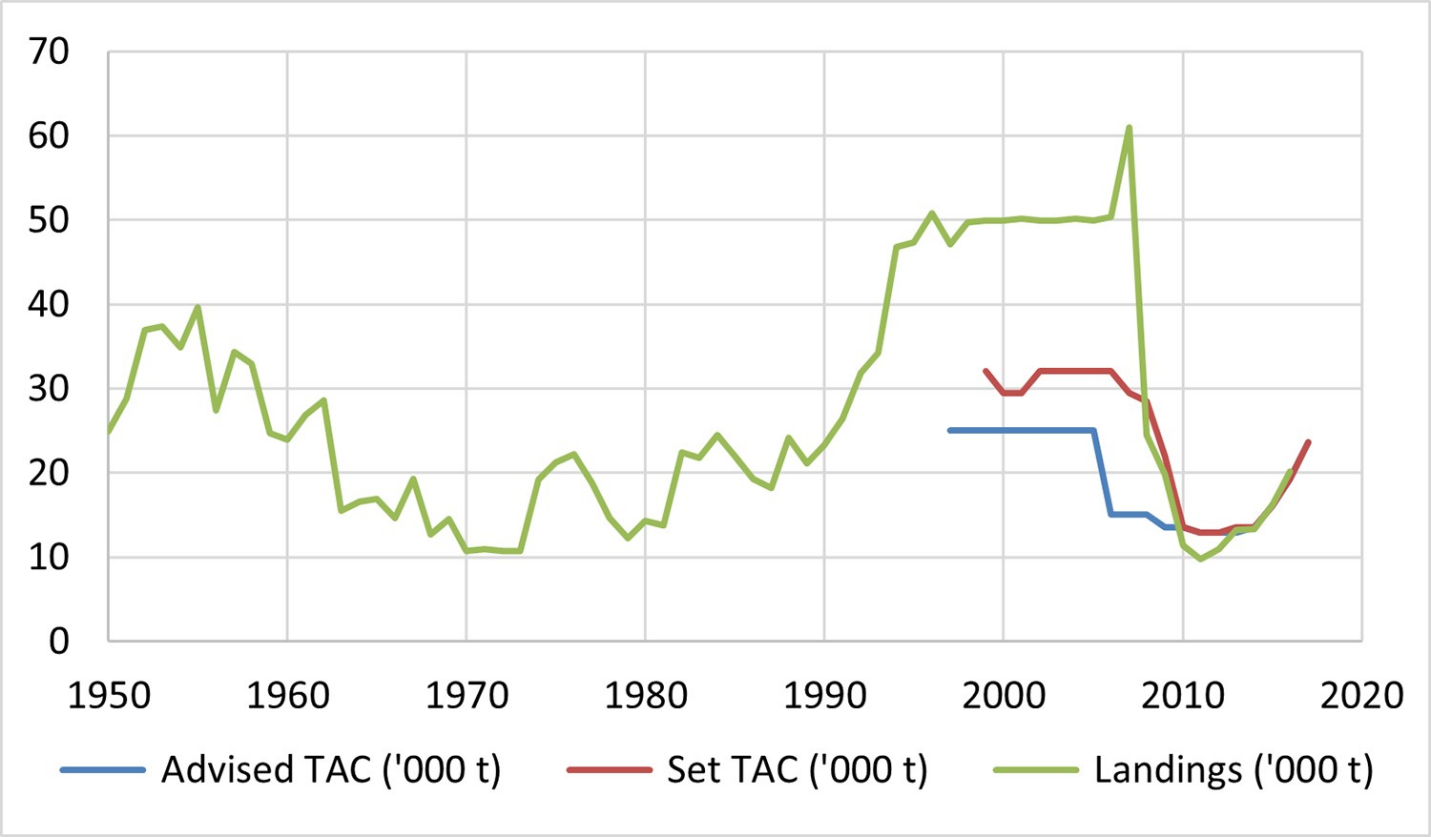
Advised TAC, set TAC and landings [36].

As shown in Fig 3, the initial established TACs exceeded scientific recommendations, and there was low compliance with it, with biological data revealing a prolonged situation of overexploitation. The continuing stock decline raised concerns over industry and institutions, and in the late 2000s support for the managing plan raised in the industry and landings adjusted to TACs. Shortly afterwards, established TACs adjusted to scientific recommendation, and now the latest qualitative assessment indicates a rapid recovery of the stock, reaching the MSY reference point [36].

**Fig 4:**
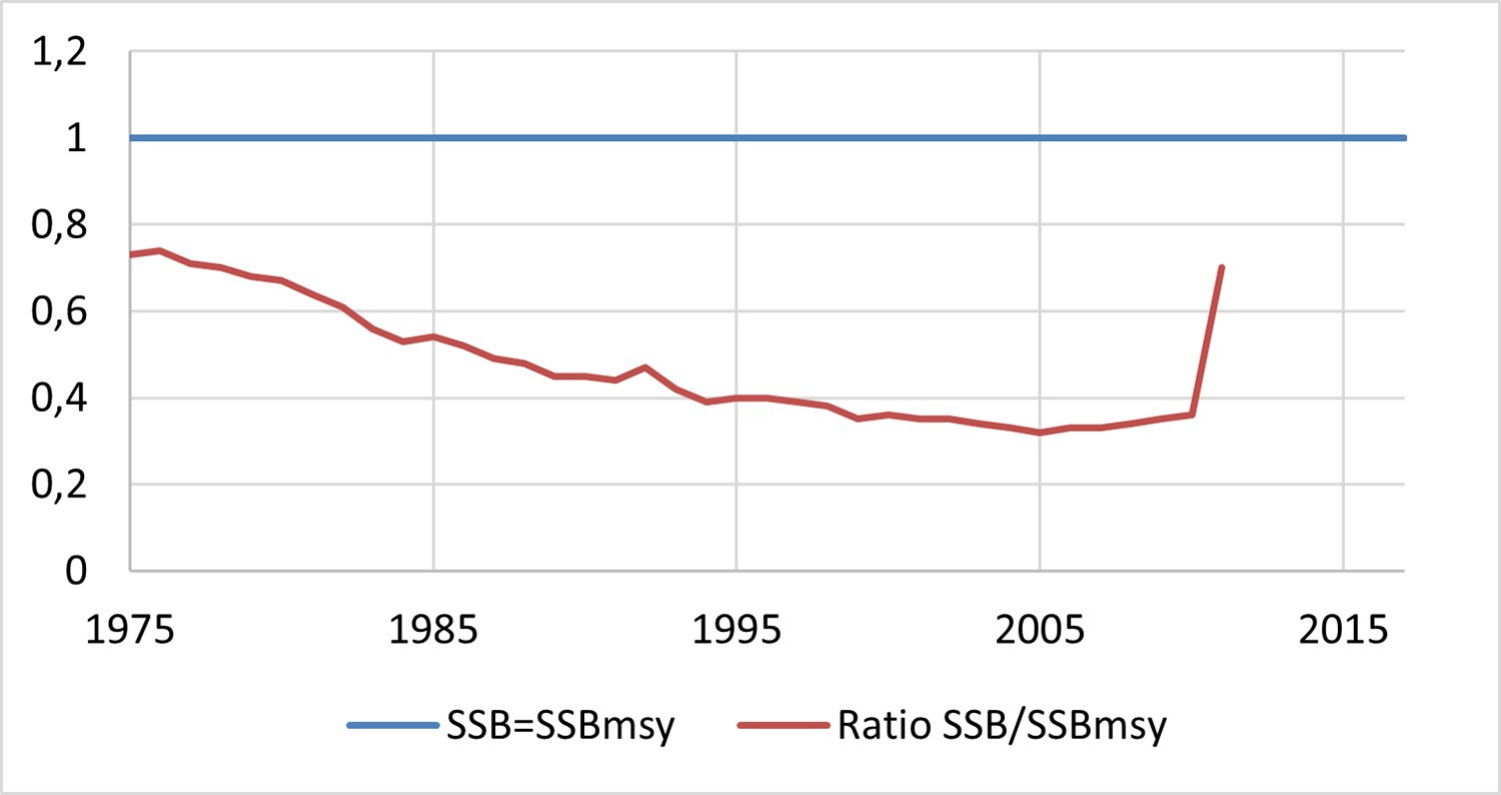
Stock biological status [36].

Fig 4 shows the relationship between spawning stock biomass (SSB), or how many fish able to reproduce live in the stock, and the spawning stock biomass at maximum sustainable yields (SSB_MSY_), the SSB required for the long-term sustainability of the stock. The ratio acts as a measure of stock health, comparable with the management goal, the maintenance of the SSB at MSY level. If the ratio is equal or higher than one, the stock is exploited at sustainable levels; if the value is lower than one, it is considered overexploited.

As shown in Fig 4, stock health has been declining since 1975, illustrating a long history of unsustainable exploitation. From 2010, with the said alignment of management, scientists and fishermen’s goals, a certain recovery of the stock is observed. The latest qualitative assessments point to an effective recovery of the stock, being exploited above MSY limits.

### 4.2 Impact of overexploitation on the market

#### 4.2.1 Trends on prices and quantities by country

In order to understand the behaviour of our series, Table 3 shows their main descriptive statistics.

**Table 3:** Main descriptive statistics.

	*Mean*	*Median*	*Standard deviation*	*Kurtosis*	*Skewness*	*Minimum*	*Maximum*
***Status***	*0*.*38*	*0*.*35*	*0*.*08*	*14*.*4*	*3*.*64*	*0*.*32*	*0*.*7*
***Quantity Spain***	*451*.*56*	*392*	*287*.*28*	*1*.*67*	*1*.*19*	*121*	*1*,*226*
***Quantity USA***	*218*.*17*	*171*	*186*.*56*	*6*.*37*	*2*.*32*	*46*	*828*
***Quantity France***	*94*.*78*	*76*.*5*	*88*.*79*	*2*.*39*	*1*.*54*	*6*	*343*
***Quantity Italy***	*319*.*89*	*177*.*5*	*355*.*25*	*4*.*09*	*2*.*11*	*20*	*1*,*332*
***Quantity Japan***	*12*,*174*.*78*	*13*,*049*	*2*,*837*.*81*	*1*.*87*	*-1*.*56*	*4*,*776*	*15*,*354*
***Quantity S*. *Korea***	*821*.*56*	*756*.*5*	*434*.*66*	*4*.*78*	*1*.*64*	*95*	*2*,*157*
***Price Spain***	*6*.*12*	*4*.*79*	*3*.*62*	*1*.*36*	*1*.*38*	*2*.*7*	*15*.*7*
***Price USA***	*14*.*83*	*15*.*12*	*8*.*6*	*-0*.*87*	*0*.*06*	*2*.*36*	*30*.*71*
***Price France***	*6*.*41*	*5*.*54*	*2*.*01*	*-0*.*21*	*1*.*08*	*4*.*39*	*10*.*52*
***Price Italy***	*4*.*94*	*4*.*24*	*2*.*16*	*-0*.*37*	*0*.*88*	*2*.*48*	*9*.*25*
***Price Japan***	*6*.*87*	*6*.*09*	*3*.*57*	*-0*.*52*	*0*.*84*	*3*.*03*	*13*.*9*
***Price S*. *Korea***	*18*.*4*	*17*.*66*	*5*.*81*	*3*.*06*	*1*.*56*	*9*.*26*	*34*.*24*

As presented in Table 3, the variable of status, constant for all countries, shows low variability over time, with a mean close to its minimum. Meanwhile, in terms of market quantities, Japan -and to a lesser extent South Korea- have the highest numbers in terms of mean values, maxima and variability. It must be mentioned that those two countries are not only two of the major global tuna importers, but also have relevant fishing activities both in the Atlantic-Mediterranean and in the Pacific stock. In terms of prices, S. Korea and the USA show the biggest means, maxima and dispersion.

**Fig 5:**
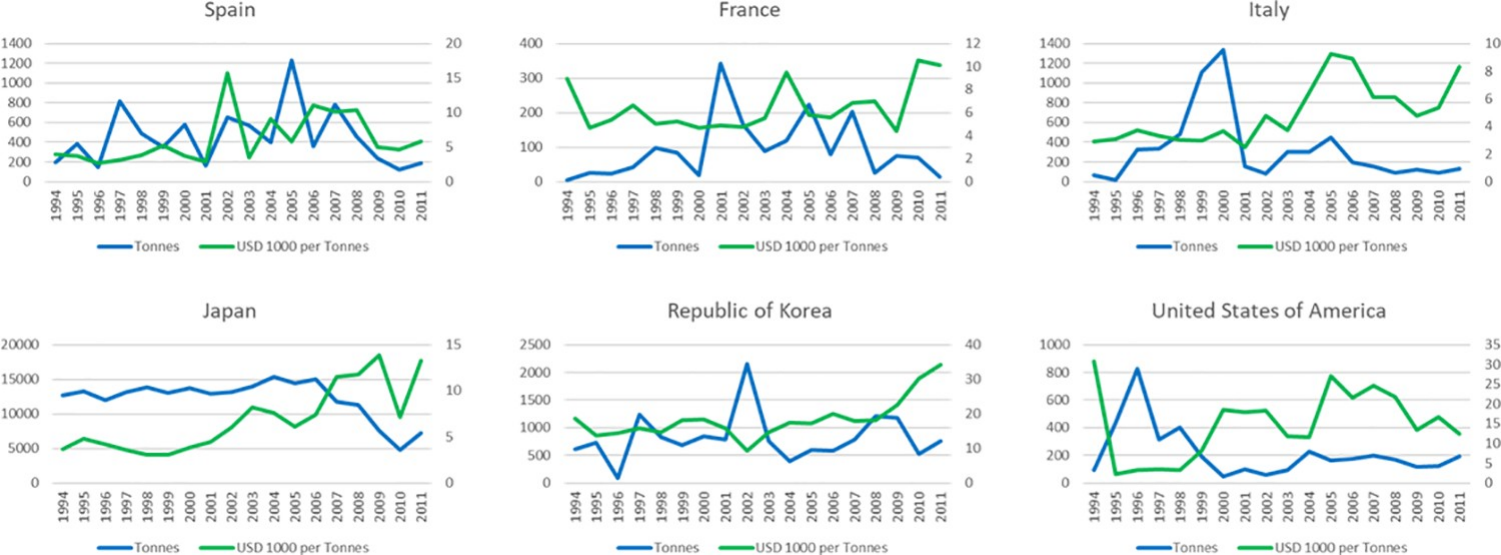
Time series evolution. Source: Own elaboration based on [34].

Fig 5 depicts the different time series used in the analysis. With few exceptions, we observe rising tendencies in prices, and declining quantities.

In several series we observe the effects of the start of the TAC system in the early 2000s, in such a way that its stricter application and its hardening to conform to scientific advice around 2008 are evident. While countries like France and Italy easily reflect the foregoing events, others like Spain and Japan manage certain stability until the observed breaks. In the USA and S. Korea, their use of the Western stock and of Pacific bluefin tuna could have a mitigating effect.

#### 4.2.2 Structural breaks detection

An overexploitation phenomenon may be revealed by an abrupt reduction of market quantities and increase in prices [17]. Therefore, structural change in the series would serve as an indicator of overexploitation trends on markets. Certain events like the increasing overexploitation, start of regulation around 2000 or the alignment of TACs with scientific advice around 2007 would be mirrored in the series, serving as symptoms of the overexploitation trends. If breakpoints were detected, they should be included in the regression analysis.

We resort to the Bai [44] and Bai & Perron [45, 46] procedure. These authors go beyond other tests for the same purpose in that they allow for multiple breakpoints that do not have to be determined a priori, that is, they are unknown. In this work we use the category of tests that uses the global maximiser for the breakpoints (the authors have also checked with two additional categories and the results do not vary); a maximum of three breaks is considered. It is important to highlight that we only consider changes in levels of the variables. In addition, and throughout our study, we use the traditional logarithmic transformation of the variables.

As the Bai-Perron test requires the stationarity of the series, they have been checked with the ADF-GLS test, chosen for its power when dealing with small sample sizes. All the series but the quantities for Japan past the test. For this specific series, we have followed Weideman *et al* [47] methodology, to test the validity of the found break. As these authors claim, when facing a non-stationary time series with a Bai-Perron break, the series are divided into segments around it and the ADF-GLS test is applied to evidence for stationarity. If both segments are stationary, it is confirmed that the unit root found in the series is due to the structural break, which can be reflected in a model through a dummy variable. Following this procedure, we divided the log of the quantities for Japan into two periods, 1994–2008 and 2009–2016, and we evidenced a rejection of the null hypothesis of unit root for both periods; this confirms the break point suggested by Bai-Perron in 2009.

The results obtained from the Bai-Perron tests for prices and quantities in the six countries are displayed in Table 4.

**Table 4:** Bai-Perron structural break test results for each variable and country.

	*l_price*		*l_quantity*	
*Country\Variable*	*Break date*	*F-statistic*	*Break date*	*F-statistic*
		*(critical value)*		*(critical value)*
***France***	*2010*	*23*.*2203 (8*.*58)*	*-*	
***Italy***	*2004*	*45*.*8701 (8*.*58)*	*-*	*-*
***Japan***	*2003*	*22*.*7003 (8*.*58)*	*2009*	*138*.*9478 (8*.*58)*
***S*. *Korea***	*-*	*-*	*-*	*-*
***Spain***	*2002*	*11*.*6418 (8*.*58)*	*-*	*-*
***USA***	*-*	*-*	*-*	

*Notes*: Critical values from Bai-Perron [45]. *l* denotes logarithm.

Following these results, there is evidence of breakpoints regarding prices and quantities. The breaks are centred around two major events. Firstly, Italy, Japan and Spain started to experience upward tends in prices in the 2002–2004 period, as a response to the scarcity generated by increased exploitation in the 90s and the introduction of a TAC system in 1999. Secondly, Japan’s increasing market quantities and France’s decrease in prices starting in the 2009–2010 period, pointing to the stock recovery trend started around 2008 due to TACs and landings adjusting to scientific recommendations. These breakpoints and trends reveal that there are two clearly differentiated phases. The first one reflects the existence of a risk of overexploitation in the fishery; the second phase poses a recovery thanks to the alignment of objectives and scientific advice.

### 4.3 Panel data estimation

A panel regression analysis has been set up in order to quantify the impact of stock health on the market. To estimate the model, the existence of unit roots in the series must be tested. We perform the test derived by Levin, Lin & Chu [41], named Levin-Lin-Chu (LLC) test. In this work we consider a constant as the deterministic component and we set a maximum of two lags. As shown in Table 5, the existence of unit roots can be rejected for both quantity and prices (the p-value for the former is much lower than for the latter); in the case of status, as it is a constant variable for all countries, the test is not performed.

**Table 5:** Levin-Lin-Chu test results.

*Variable*	*t-adjusted*	*p-value*
*l_price*	*-2*.*02539*	*0*.*0214*
*l_quantity*	*-2*.*87381*	*0*.*0020*

All the variables (up to two lags in quantity and status) have been initially introduced in the model estimation. We accounted for breakpoints by including a dummy variable that equals 1 for the break dates and countries shown in Table 4, and zero in the remaining cases. The model is subject to further refinement, so that non-significant coefficients are successively excluded to conserve degrees of freedom. The final estimated model is reported in Table 6.

**Table 6:** Panel data regression model.

*Variable*	*LSDV model*
*Constant*	*-1*.*4743 (-1*.*9816)* *
*l_quantity*	*-0*.*1379 (-2*.*1954)* **
*l_quantity (t-1)*	*-0*.*1359 (-2*.*2006)* **
*l_status*	*0*.*4806 (1*.*7249)* *
*l_status (t-1)*	*-5*.*3450 (-7*.*9029)* ***
*Dummy break*	*0*.*6620 (3*.*2453)* ***
*R* ^*2*^	*0*.*7475*
*Adjusted R* ^*2*^	*0*.*7198*
*F-statistic*	*26*.*9444* ***
*Durbin-Watson statistic*	*1*.*2326*
*Breusch-Pagan LM*	*17*.*3124*

Notes. Values between parentheses are t-statistics. Symbols

*, ** and *** denote 10%, 5% and 1% significance levels, respectively.

The model points to the individual significance of all the variables included, and most of them are relevant at 1% and 5% significance levels. Following the estimated model, the major weight of trade quantities and stock status in explaining the behaviour of prices should be highlighted. As expected, market quantities are negatively related to prices, and the effect of quantities in the current year is similar to the effect of previous years’ quantities. Thus, an increase (decrease) of 1% in the current or the previous period quantity generates a 0.14% decrease (increase) in prices. This fact could hint that the market also uses previous years’ quantities to estimate expected abundancy when setting prices.

Regarding the status, the relevance of its recent history is also clear from the results. Thus, we observe that a 1% improvement in the current status of the stock could reduce next year prices by 5.35%. However, the effect of actual stock status is not clear, as the associated coefficient does not show the expected sign. As its effect is relatively insignificant compared with its first lag, it seems that prices are mainly affected by the health of the stock in the previous year–which makes sense, as past spawning biomass builds current biomass.

As it is observed, the different breakpoints play an important role in the relationship under study; their impact on prices is direct or positive, considering the value of the associated coefficient. Two main events are reflected in this relationship. Firstly, the deterioration of the health of the stock caused by an increase in fishing pressure in the 1990s and, secondly, the regulatory reaction in 1999. The first result comes from the evidence of a decreasing trend in quantities and increasing in prices. The second one relates to the improvement of the health of the stock thanks to the alignment of the landings, the TACs and the scientific advice from 2007. The results of the estimated model demonstrate the existence of individual differences not only between countries, but also over time.

**Fig 6:**
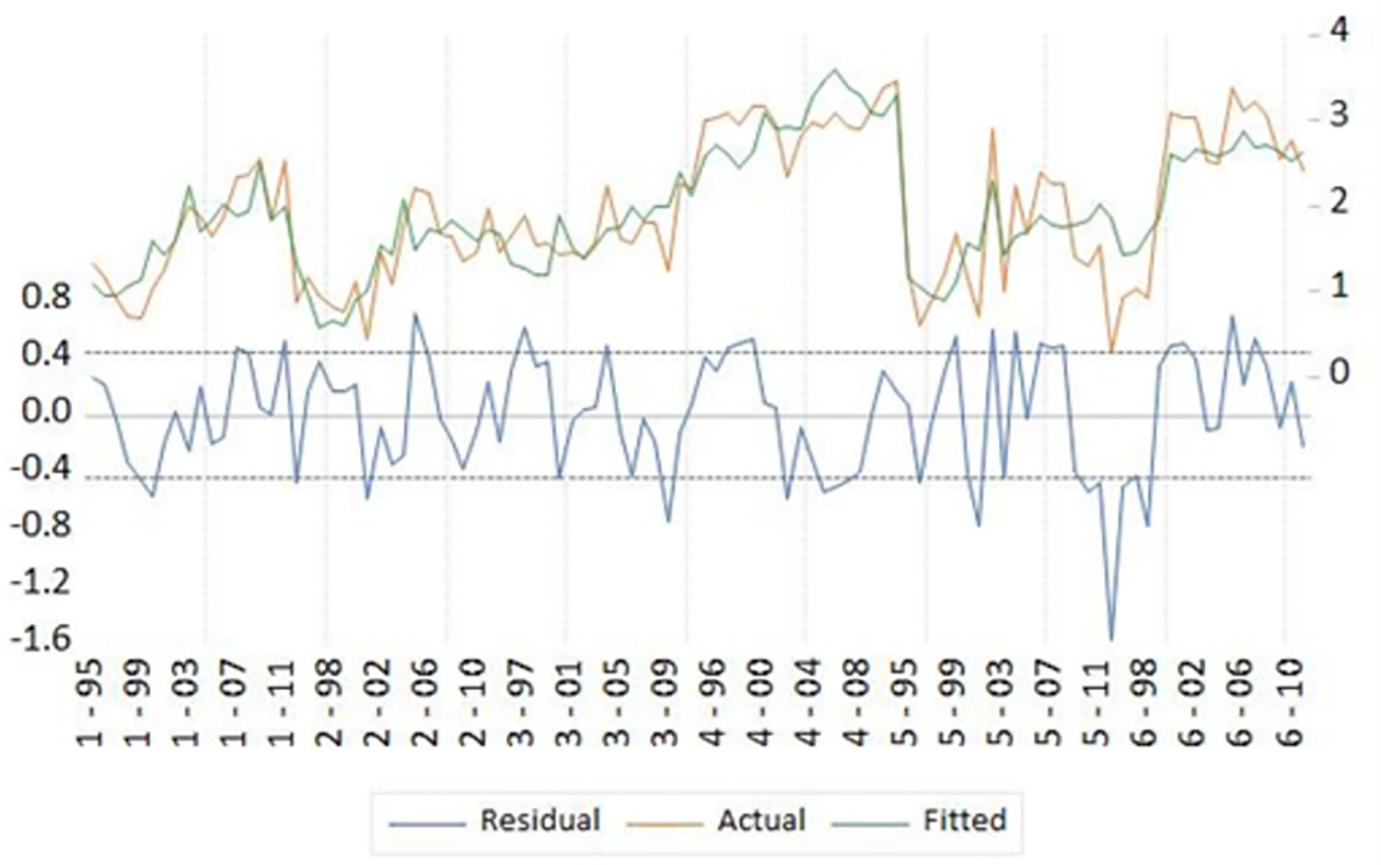
Residuals, actual and fitted values for l_price from the estimated model.

Fig 6 displays the estimated residuals from the model together with the actual and fitted values of the dependent variable, the price.

Diagnostic and evaluation statistics point to a satisfactory panel model. The variables are individually and jointly significant, the model shows a good fit, as 75% of the sample variability of prices is explained by the model, and there is no cross-section dependence in the residuals. The common diagnostic statistics have been checked and are in the correct intervals, like Durbin-Watson or Breusch-Pagan, among others, so to the best of our knowledge the model, pointing to a good estimation of the model.

## 5 Conclusions

The increased fishing pressure in the last decades of the twentieth century raised great concern about the overexploitation of Atlantic bluefin tuna. This fact can be confirmed by examining the structural changes in the historical series analysed in different countries. To keep biomass close to the Maximum Sustainability Yield (MSY), a quota management system was established at the end of the 1990s, although unaligned with the scientific recommendations and with low compliance from the industry. Around 2005, the situation began to change. Although the constraints became even stricter, in 2010 catches, TACs and scientific advice were aligned, which allowed a recovery path to begin. This confirms the importance of coherent goals and the need to integrate stakeholders to have a successful management strategy.

In 2010, the improvement in the health of the stock was reflected in some markets with increases in quantities and decreases in prices. The estimated panel data model confirms the negative relationship between stock health and prices, revealing stock management as an effective measure to increase the availability and affordability of the resource. Following our estimations, 1% increases in stock health translate into 5% reductions in prices, proving that rebuilding and conservation policies are effective tools for ensuring food accessibility.

There are two main policy implications that can be drawn from this study. Firstly, derived from our main analysis, we find that introducing biological variables in economic models can help policy maker to better evaluate the impact of policies in the management of the stocks, and the implications of this policies in terms of food security though affordability. Prior studies have described the threat to food security and economic equality caused by rising fish prices due to overexploitation [48, 49]. In this study we develop a methodology to measure its impact and recovery potential. Secondly, in our descriptive and breakpoint analysis of the recent history of Atlantic bluefin tuna, we find an example of the benefits of cooperation between industry and public administrations to set up control mechanisms based on scientific recommendations. With more than twenty countries involved in the fishery, the case of bluefin tuna shows the importance of cooperation among stakeholders to recover and ensure the sustainability of the resource.

The present study also contributes to the fish price literature by adding a new kind of variable to price modelling. As exposed in the introduction, price models use a wide range of variables like quantities [8–10], type of gear [9, 11] or consumer preferences [14, 15], among others. Following prior authors who used ecological-related variables in economic models [18], we propose and successfully test the addition of biological variables that reflect the sustainability of the stock, by adding them to a price model that depends upon quantities. Future research could implement this type of variables in more complex fish price models, together with variables like gear type or economic cycle, to give a more complete picture of the dynamics of prices and test the behaviour of the relationship price-sustainability under different exploitation and/or economic scenarios. Another interesting avenue of research could be to explore the relationships we have found by testing other biological/exploitation variables. An example could be to use the Maximum Economic Yield instead of Maximum sustainable Yield, which is not as accessible but can be found for some fisheries, to explore the efficiency dimension of sustainability.

## Supporting information

S1 Data (XLSX)
